# Population genomics of Brazilian native fruit species of *Eugenia* spp. (Myrtaceae) for conservation and improvement

**DOI:** 10.3389/fpls.2025.1670349

**Published:** 2026-01-12

**Authors:** Laecio Fernandes Souza Sampaio, Maria Imaculada Zucchi, Carlos Augusto Colombo, Angelo Pedro Jacomino, Antonio Figueira, Francisco de Assis Alves Mourão Filho

**Affiliations:** 1 Escola Superior de Agricultura Luiz de Queiroz, Universidade de São Paulo, Piracicaba, SP, Brazil; 2 Polo Regional de Desenvolvimento Tecnológico do Centro Sul, Agência Paulista de Tecnologia dos Agronegócios, Piracicaba, SP, Brazil; 3 Instituto Agronômico de Campinas, Centro de Recursos Genéticos Vegetais, Campinas, SP, Brazil; 4 Centro de Energia Nuclear na Agricultura, Universidade de São Paulo, Piracicaba, SP, Brazil

**Keywords:** Atlantic forest, *E. brasiliensis*, *E. involucrata*, *E. pyriformis*, genetic markers, K-means, SNP

## Abstract

Brazil is a global biodiversity hotspot, especially in the Atlantic Forest biome, which contains a high diversity of native fruit species that remain underutilized and understudied. Native fruit trees, particularly those in the Myrtaceae family, have great potential to become new fruit crops contributing to food security. The genus Eugenia encompasses several native species that have been little investigated, including *Eugenia brasiliensis* Lam. (grumixama), *E. pyriformis* Cambess (uvaia), and *E. involucrata* DC (Rio Grande cherry). This study investigated the genomic diversity and structure of several populations of these three native fruit species using single-nucleotide polymorphism (SNPs) markers obtained through genotyping by sequencing. We analyzed 73 accessions of *E. brasiliensis*, 93 of *E. pyriformis*, and 62 of *E. involucrata*, derived from three, four, and seven populations, respectively, maintained as living collections (due to their desiccation-sensitive seeds) in research institutions, urban afforestation projects, and small rural properties in the states of São Paulo and Minas Gerais, Brazil. The comparison among *E. brasiliensis*, *E. pyriformis*, and *E. involucrata* accessions revealed 2,299, 2,872, and 1,471 SNPs, respectively. These markers effectively characterized each species’ genomic diversity and population structure, revealing levels of diversity (*He* = 0.22, 0.19, 0.21 for grumixama, uvaia, and Rio Grande cherry respectively) and inbreeding (*f* = -0.06; 0.05; -0.04, respectively) consistent with their respective mating biology. Significant genetic structure was detected between collections (*Phi_ST_* = 0.29; 0.10; 0.23 for *E. brasiliensis*, *E. pyriformis*, and *E. involucrata*, respectively), confirmed by discriminant and principal component analyses, indicating an important diversity between and within the collections. The data will serve to identify the most divergent accessions to help prioritize accessions for fruit quality assessments and for conservation, while identifying parents to guide hybridizations to initiate a breeding program. The study highlights the importance of employing population genomics approaches to develop improved management practices for these fruit species, ultimately promoting the conservation and valorization of Brazilian native genetic resources.

## 1 Introduction

Brazil is a recognized global biodiversity hotspot, particularly in the Atlantic Forest biome, which harbors an exceptional wealth of native fruit species that have mainly remained underutilized, scientifically neglected, and poorly integrated into sustainable agricultural or economic systems (Araújo et al., 2019). Native fruit species represent a potential genetic resource to integrate production systems as new crops, which can contribute to food security (Souza et al., 2018). The genus Eugenia, Myrtaceae, for instance, contains numerous native Brazilian fruit species from this biome, most of which are poorly characterized. Our interest focused on *Eugenia brasiliensis* Lam. (grumixama.), *E. pyriformis* Cambess (uvaia), and *E. involucrata* DC. (Rio Grande cherry), which are highly valued for their flavor and pulp nutritional properties (Araújo et al., 2019). These Eugenia species have been subjected to studies regarding their fruits’ nutritional and agro-industrial characteristics (Flores et al., 2012; Infante et al., 2016; Sardi et al., 2017; Lazarini et al., 2018; Silva et al., 2019; Soares et al., 2019; Xu et al., 2020; Nehring et al., 2022; Sganzerla et al., 2022). Their fruits contain high levels of vitamins, minerals, antioxidants, together with other bioactive compounds (*e,g.*Silva et al., 2022; Spricigo et al., 2023), and display traditional uses in various food and popular medicinal applications (Araújo et al., 2019; Schmidt et al., 2019; Farias et al., 2020).

Therefore, we have selected these Eugenia species as they are promising new crops in Brazil, due to their desirable fruit sensorial and nutritional qualities, and their adaptability to local climates (Araujo et al., 2024; Saliba et al., 2025). However, little is known about their genetics and mating biology. The region of natural dispersion and center of diversity of these species have not been determined and there is little information about areas of natural occurrence. However, it is well established that the Brazilian biomes where anecdotal references to their occurrence have been reported have been continually threatened by land-use change, urbanization, and deforestation (Araujo et al., 2024). In emerging crops and in the process of domestication, as is the case of these *Eugenia* species, knowledge of genetic diversity and population structure is crucial for the development of strategies to sustainably exploit these species and to preserve genetic resources, particularly for these species that display desiccation-sensitive seeds, which largely limit the long-term conservation of accessions (Delgado and Barbedo, 2012; Inocente and Barbedo, 2019), while starting a breeding program (Allendorf et al., 2013; Salgotra and Chauhan, 2023).

The rapid advancement of next-generation sequencing (NGS) technologies, along with their cost reduction, has enabled large-scale identification of single-nucleotide polymorphisms (SNPs) (Nadeem et al., 2017; Rasheed et al., 2017; Rasheed and Xia, 2019; Kumar et al., 2021). SNPs are the most abundant, codominant, and stable polymorphisms in plant genomes. The possibility of automating the detection and genotyping of these markers offers a notable advantage over previously used marker types (Morin et al., 2004; Helyar et al., 2011; Mammadov et al., 2012). Several strategies can be used to discover SNPs (Andrews et al., 2016). The ideal SNP genotyping would be whole-genome sequencing (Aguirre et al., 2024); however, its high cost favored the development of genotyping arrays and Reduced-Representation Sequencing (RRS) approaches for SNP identification (Andrews et al., 2016). The RRS methods grouped under the generic term ‘genotyping by sequencing’ (GBS) are the most cost-effective strategy currently used for non-model organisms, which combine genome reduction and sampling of both coding and non-coding regions with a genome-wide coverage, and it can be used in species lacking a reference genome (Elshire et al., 2011; Poland et al., 2012; Andrews et al., 2016; Brhane et al., 2022).

Here, we employed GBS to identify SNP markers and assess the genetic diversity and population structure of *E. brasiliensis* (grumixama), *E. pyriformis* (uvaia), and *E. involucrata* (Rio Grande cherry) accessions, which are maintained in small collections by growers and research institutions in São Paulo and Minas Gerais, Brazil. We hypothesized that there was strong genetic differentiation among collections, with clustering patterns corresponding to their geographic origins, and that individual collections would exhibit unique diversity and inbreeding profiles reflecting their specific establishment histories.

## 2 Materials and methods

### 2.1 Plant material and collection sites

**Figure 1:**
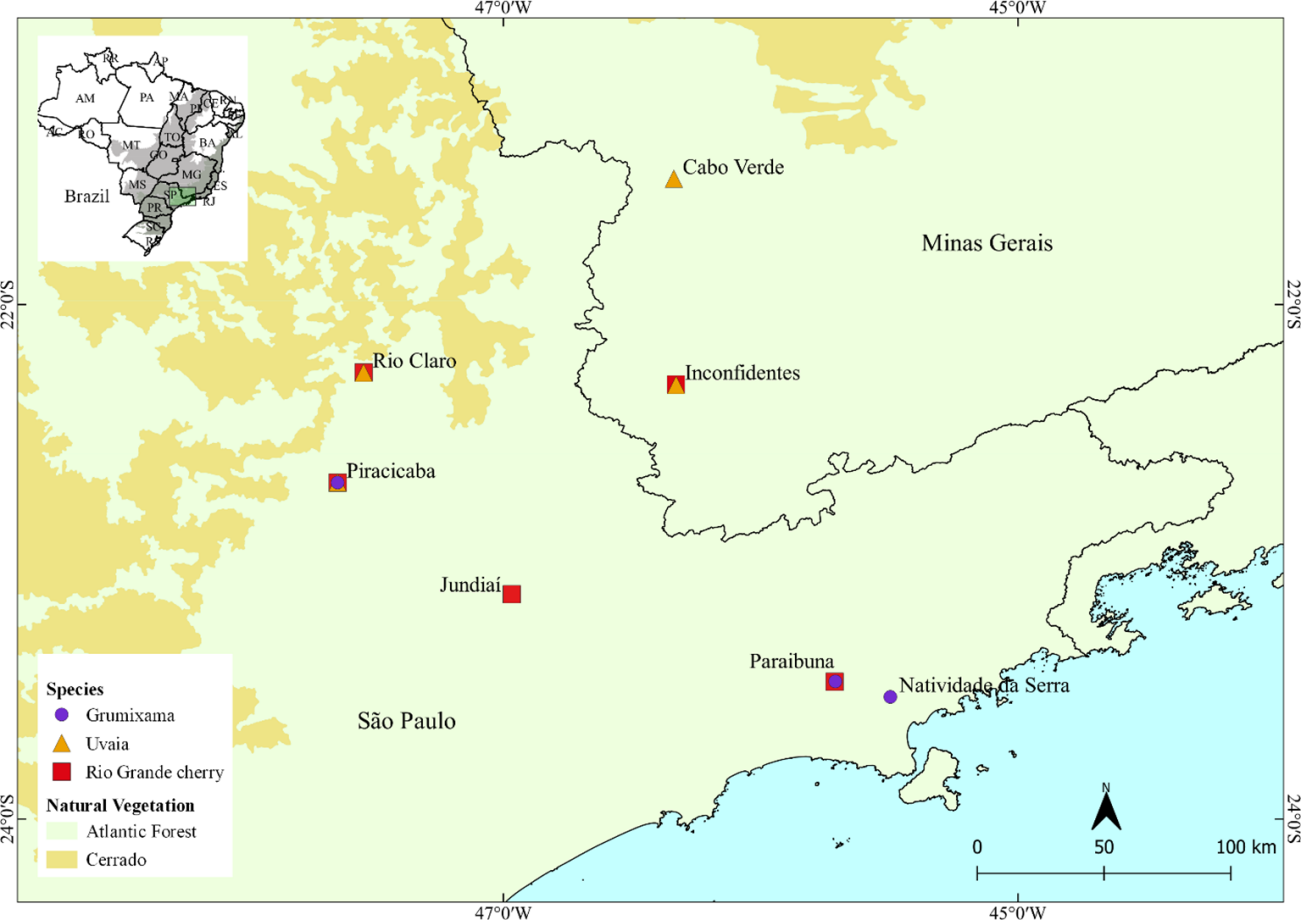
Map of collection sites for *Eugenia brasiliensis* (grumixama), *E. pyriformis* (uvaia) and *E. involucrata* (Rio Grande cherry) accessions in the Atlantic Forest regions of São Paulo and Minas Gerais states. Author: Laecio Sampaio, 2020 - QGIS.

Accessions of *E. brasiliensis* (grumixama - *n* = 76), *E. pyriformis* (uvaia – *n* = 111) and *E. involucrata* (Rio Grande cherry –*n* = 69) were originally collected in several municipalities in São Paulo and Minas Gerais, Brazil (**Figure 1**), and were kept as living specimens (accessions) on private properties of small growers, collectors of fruit species, University collections, and experimental stations (**Supplementary Table 1**). Accessions of *E. brasiliensis* were collected in three locations (municipalities), while accessions of *E. pyriformis* were collected in four locations. Samples of *E. involucrata* were gathered from seven locations in five municipalities (**Figure 1**, **Supplementary Table 1**). Leaf samples from each of the three species’ accessions were collected, wrapped in aluminum foil, and stored in a thermal box containing ice until arrival to the laboratory, where they were kept at – 80°C until DNA isolation. The initial sampling included 76 accessions of *E. brasiliensis*, 111 accession of *E. pyriformis*, and 69 of *E. involucrata*. After quality filtering the sequenced reads (see below), individuals with more than 40% missing data or duplicated genotypes were removed, resulting in a final dataset of 73, 93, and 62 accessions used in the analyses for *E. brasiliensis*, *E. pyriformis*, and *E. involucrata*, respectively.

### 2.2 Extraction of genomic DNA

Genomic DNA extraction was performed using 50 mg of leaf tissue (Sereno et al., 2006). DNA quality was assessed by electrophoresis (1 V cm^-1^) in a 1% agarose gel stained with SYBR gold (Thermo Fisher Scientific Inc.; Waltham, MA, USA) and quantified by fluorescence using the Qubit dsDNA BR assay (Thermo Fisher Scientific). The DNA concentration of the samples was standardized to 25 ng μL^-1^.

### 2.3 Preparation and sequencing of GBS libraries

The genomic libraries were prepared according to the protocol of Poland et al. (2012). For each sample, approximately 175 ng of genomic DNA (7 μL) was digested at 37°C for 12 h with *Nsi*I and *Mse*I to reduce sequence complexity (Poland et al., 2012). After digestion, the fragments were ligated to 0.02 μM specific Illumina adapters (Illumina Inc.; San Diego, CA, USA), which are complementary to the restriction sites. At this stage, each sample received an adapter containing barcode sequences to identify each individual after sequencing. The ligation was carried out for two h at 22°C, followed by 20 min at 65°C. The ligation reaction products from each sample were then mixed into a single pool and purified with the QIAquick PCR purification kit (Qiagen; Germantown, MD, USA). The libraries were subjected to amplification. For each library, eight replicates were made, each containing 10 μL of purified and amplified ligation, using 12.5 μL of Phusion High-Fidelity PCR Master Mix NEB (New England Biolabs Inc.; Ipswich, MA, USA) and two μL of Illumina forward and reverse primers (10 μM), in a final volume of 25 μL, using the following amplification program: 95°C for 30 s, followed by 16 cycles of 95°C for 10 s, 62°C for 20 s, 72°C for 30 s, finishing at 72°C for 5 min. Then, the libraries were purified again using the QIAquick PCR Purification Kit. Before sequencing, the average size of the DNA fragments was verified using the Agilent DNA 12000 kit and the 2100 Bioanalyzer System (Agilent; Santa Clara, CA, USA). The libraries were quantified by quantitative PCR (qPCR) in a CFX 384 thermocycler (Bio-Rad, Hercules, CA, USA) using the KAPA Library Quantification kit (KAPA Biosystems, Wilmington, MA, USA). Finally, the libraries were normalized and diluted to 1.8 pmol μL^-1^ and sequenced in the *single-read* configuration and 150 base pairs, using the Illumina NextSeq 500/550 Mid Output kit v2.5 (150 cycles), on the NextSeq550 platform (Illumina). We have sequenced replicates of DNA for a sample of accessions as part of quality control.

### 2.4 Genotyping and filtering of SNPs

Read sequence quality was preliminarily checked by FastQC (Andrews, 2010). Subsequently, demultiplexing and quality filtering were performed using the process_radtags module based on the STACKS v.2.437 pipeline (Catchen et al., 2013). Quality filtering was performed with the default setting, and sequences were truncated to 100 nucleotides. Next, reads with adapter sequences were removed, allowing for incompatibility of up to 2 bases. The barcodes of the retained sequences were removed, and SNPs were identified. All the species investigated here lack a reference genome; therefore, the ustacks module was used to construct all loci *de novo*. In this step, two parameter combinations were used for filtering. For *E. pyriformis* and *E. involucrata*, the parameters were: minimum depth to form a locus (-m) = 2, maximum intra-individual distance between stacks (-M) = 3, and maximum distance allowed to align secondary readings to primary stacks (-N) = -M + 2 (this being the last default configuration of the ustacks module). To build the locus catalog in the cstacks module, the maximum difference allowed between the stacks (-n) was defined as 2. Finally, the SNPs were filtered in the populations module, following the criteria: minimum depth of sequencing ≥ 3X, minor allele frequency (MAF) ≥ 0.05, filtering only SNPs that occurred in all locations/collections (four sites for *E. pyriformis*; seven sites for *E. involucrata*erry), and at least in 75% of samples from each location/collection. The parameters used for *E. brasiliensis* in the ustacks module were: -m = 3, -M = 2, and -N = 2. Subsequently, a locus catalog (cstacks) was built, allowing a maximum of 2 differences between stacks from different accessions. Then, in the *populations* module, the final filtering of SNPs was carried out with a minimum sequencing depth of 5X, MAF ≥ 0.05 and minimum occurrence in 90% of individuals in each location/collection. The filtering parameters in the *ustacks* module were optimized for each species to maximize locus construction quality based on the sequencing output. For *E. brasiliensis*, a higher average sequencing depth (16.2X) allowed for more stringent criteria (*e.g.*, minimum depth of 5X and 90% of individuals) compared to *E. pyriformis* and *E. involucrata* (6.3X and 6.1X, respectively), for which a minimum depth of 3X and presence in 75% of individuals was required. This tailored approach ensured that the final SNP dataset for each species was of the highest possible quality. The sequence data were deposited at the BioStudies repository under the ids S-BSST2086 (*E. pyriformis*), S-BSST2087 (*E. brasiliensis*), and S-BSST2088 (*E. involucrata*).

### 2.5 Genomic and population analyses

First, the data was filtered to remove loci and individuals with high rates of missing data. SNP loci with more than 20% missing genotypes were removed from the dataset using the ‘missingno’ function in poppr v.2.8.5 (Kamvar et al., 2014) in R software (R Core Team, 2021). Individuals with more than 40% missing and/or duplicate data were also removed from analyses. This initial filtering resulted in the exclusion of zero locus and 3 individuals for *E. brasiliensis*, 222 loci and 18 individuals for *E. pyriformis*, and 44 loci and 7 individuals for *E. involucrata*.

Genomic diversity was characterized by the percentage of polymorphic loci (*% P*), the number of alleles (*Na*), the number of alleles per locus (*A*), the number of private alleles (*Pa*), allelic richness (*AR*), and observed (*Ho*) and expected (*He*) heterozygosities, calculated based on Nei’s (1978) gene diversity metric. The fixation indices (*f*) were also estimated, and their confidence intervals were obtained with 1000 bootstraps. Genomic diversity and fixation indices were estimated using the diveRsity (Keenan et al., 2013) and poppr (Kamvar et al., 2014) packages in R software (R Core Team, 2021).

Genetic differentiation between accession groups was estimated using pairwise *PhiST* and confidence intervals with 1000 bootstraps, as implemented in the Hierfstat package (Goudet, 2005) within R (R Core Team, 2021). Two methods were used to explore the population structure of the species, both from the Adegenet v.2.1.2 package (Jombart and Bateman, 2008) in R. First, for each species, a principal component analysis (PCA) was performed using the *dudi.pca* function. Second, a Discriminant Principal Component Analysis (DAPC) was conducted (Jombart et al., 2010). For each species, the groups were *de novo* identified by the K-means clustering algorithm, from the *find.clusters* function after data transformation by Principal Component Analysis (PCA). K-means was performed with K ranging from 1 to 40, and Bayesian Information Criterion (BIC) was used to determine the most appropriate value of K (**Supplementary Figures S1**-**S3**). Individuals were assigned to groups using DAPC. To avoid retaining too many dimensions in the discriminant analysis (DPCA), the optimal number of PCs was determined using the *optim.a.score* function. Based on this analysis, 5 PCs were retained for *E. brasiliensis*, 7 for *E. pyriformis*, and 6 for *E. involucrata*. The probability of adherence of individuals from each collection to each group was determined after DAPC (subsequent assignment of DAPC analysis).

### 2.6 Core collection

The R package CoreHunter 3.0 (De Beukelaer et al., 2018) was used to assemble a nuclear collection that represented the maximum genetic diversity of the total number of accessions collected for *E. brasiliensis*. We used the Entry-to-nearest-entry (E-NE) method, implemented in the CoreHunter 3.0 using the Modified Roger’s distance (De Beukelaer et al., 2018). Several subsamples were generated, adjusting the size of the desired core collections to identify a subset of genotypes that could capture maximum allele diversity. Collection sizes ranged from 10 to 30% for all datasets. In each collection, genetic diversity parameters and a principal coordinate analysis (PCoA) based on the standard-covariance matrix were determined using GenAlEx v. 6.5 (Peakall and Smouse, 2012).

## 3 Results

### 3.1 Overall SNP discovery in *Eugenia*

The GBS libraries were successfully sequenced for 76 *E. brasiliensis* (grumixama) accessions, 111 *E. pyriformis* (uvaia)accessions, and 69 *E. involucrata* (Rio Grande cherry) accessions. The GBS libraries of grumixama, uvaia, and Rio Grande cherry generated a total of 127,073,304, 101,022,119, and 100,281,548 reads, respectively. After quality control and filtering, the total number of accessions retained was 73 for *E. brasiliensis*, 93 for *E. pyriformis*, and 62 for *E. involucrata*, and the total number of reads retained was 122,831,851 for grumixama, 59,363,247 for uvaia, and 62,784,718 for Rio Grande cherry. A total of 2,299 SNPs was identified for *E. brasiliensis* (with an average depth per sample equal to 16.2X); 2,872 SNPs for *E. pyriformis* (with an average depth per sample equal to 6.3X); and 1,471 SNPs for *E. involucrata* (with an average depth per sample equal to 6.1X).

### 3.2 Population genomic analysis of *E. brasiliensis*

We utilized the 2,299 SNP markers identified by GBS to assess the genetic diversity and population structure of the 73 *E. brasiliensis* accessions from the three collections. The genetic diversity (*He*) ranged from 0.21 to 0.24, with an average of 0.22 among the grumixama collections (**Table 1**). The Paraibuna collection displayed the largest genetic diversity (*He* = 0.24), a higher percentage of polymorphic loci (%*P*), a greater number of alleles (*Na*), and a higher number of private alleles (*Pa*). The Piracicaba and Natividade da Serra collections presented identical *He* values (0.21). The observed heterozygosity (*Ho*) ranged from 0.22 (Piracicaba) to 0.30 (Natividade da Serra), with an average of 0.25. The Natividade da Serra collection had *Ho* greater than *He*, presenting a significant excess of heterozygotes (*f* = -0,31). The Piracicaba and Paraibuna collections showed low fixation indices (*f* = -0.04 and 0.05, respectively), which were not significantly different from zero, suggesting that these populations were in equilibrium.

**Table 1:** Estimates of genomic diversity and inbreeding of accessions of *Eugenia brasiliensis* (grumixama), *E. pyriformis* (uvaia), and *E. involucrata* (Rio Grande cherry).

*E. brasiliensis*									
Collections	N	%P	Na	A	Pa	Ho	He	f	f (IC 95%)
Natividade da Serra	11	61.07	1.61	1.37	90	0.30	0.21	-0.31	- 0.50/-0.31
Piracicaba	15	61.94	1.62	1.36	114	0.22	0.21	-0.04	- 0.31/0.15
Paraibuna	47	84.25	1.84	1.40	394	0.23	0.24	0.05	0.00/0.11
Average		69.09	1.69	1.38	–	0.25	0.22	-0.06	–

*E. pyriformis*									
Collections	N	%P	Na	A	Pa	Ho	He	f	f (IC 95%)
Rio Claro	13	72.8	1.72	1.29	0	0.18	0.20	0.06	- 0.04/0.15
Cabo Verde	42	92.2	1.92	1.33	193	0.20	0.22	0.09	0.04/0.13
Inconfidentes	24	67.5	1.67	1.29	49	0.19	0.18	-0.08	- 0.12/- 0.04
Esalq	14	69.5	1.69	1.29	43	0.15	0.18	0.14	0.03/0.20
Média	–	75.49	1.75	1.30	–	0.18	0.19	0.05	–

*E. brasiliensis*									
Collections	N	%P	Na	A	Pa	Ho	He	f	f (IC 95%)
Piracicaba 1	20	47.0	1.21	1.19	5	0.14	0.11	-0.22	-0.37/-0.06
Rio Claro	4	62.9	1.47	1.38	2	0.23	0.23	-0.02	-0.30/0.13
Jundiaí 1	5	76.6	1.53	1.44	0	0.31	0.26	-0.16	-0.40/-0.05
Jundiaí 2	3	53.6	1.44	1.35	0	0.27	0.20	-0.34	-0.78/-0.16
Piracicaba 2	9	76.1	1.43	1.36	0	0.22	0.23	0.08	-0.20/0.19
Inconfidentes	5	64.8	1.47	1.39	2	0.21	0.23	0.06	-0.34/0.25
Paraibuna	16	79.3	1.43	1.37	3	0.20	0.23	0.09	0.00/0.21
Average	–	65.8	1.66	1.35	–	0.22	0.21	-0.04	–

The accessions of *E. brasiliensis* were sampled in three collections and analyzed using 2299 single-nucleotide polymorphism (SNP) markers. The accessions of *E. pyriformis* derived from four collections and were analyzed using 2872 SNPs. The *E. brasiliensis* accessions were sampled in seven collections and were analyzed using 1471 SNPs.

Number of individuals (N); percentage of polymorphic loci (% *P*); number of alleles (*Na*); number of alleles per locus (*A*); Private alleles (*Pa*); observed (*Ho*) and expected (*He*) heterozygosities; fixation index (*f*); *f* (95% CI) = lower and upper 95% confidence intervals of fixation indices.

**Figure 2:**
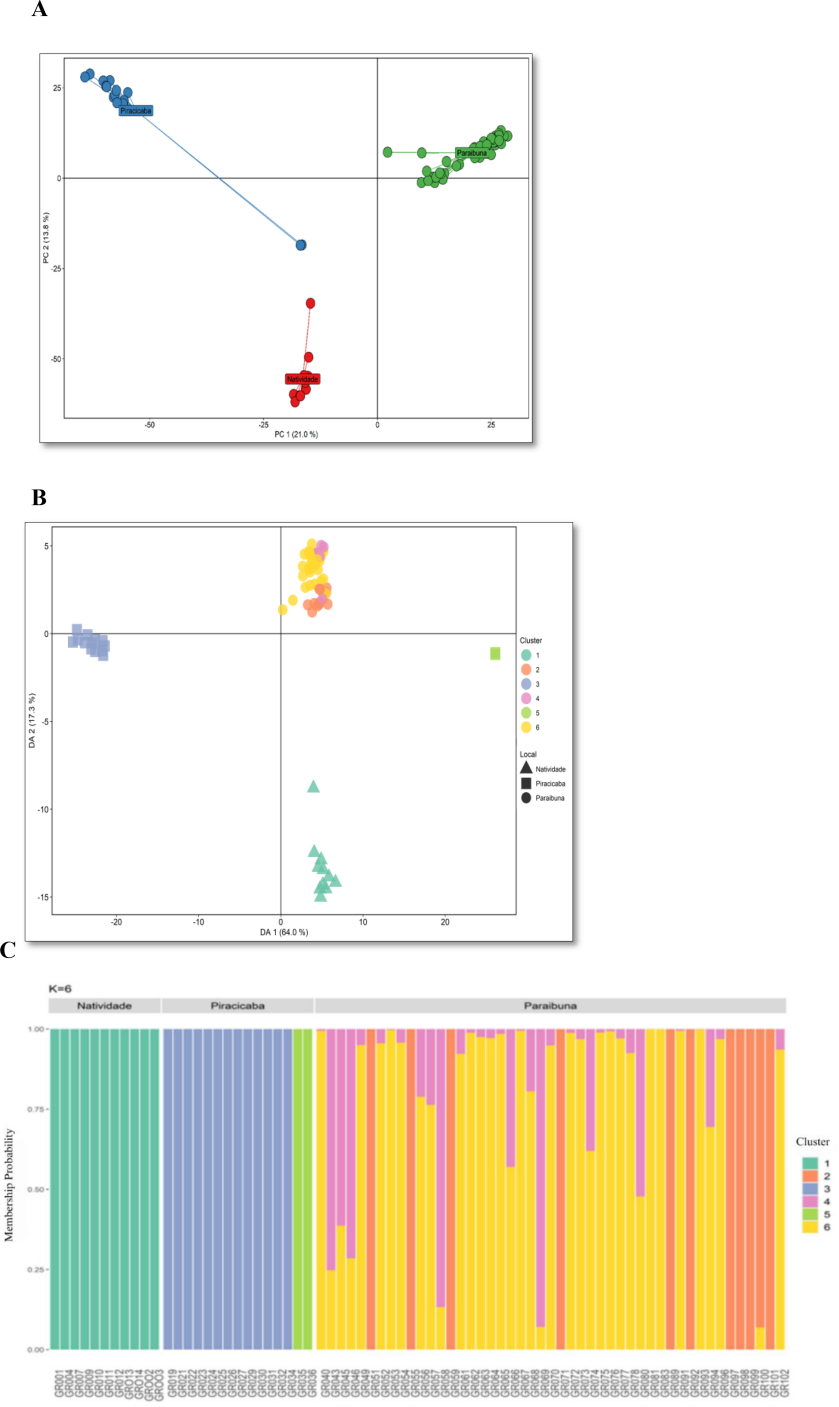
**(A)** Principal component analysis (PCA) representing the genetic structure of three *Eugenia brasiliensis* (grumixama) collections based on 2299 single nucleotide polymorphism markers. In red, individuals from the Natividade da Serra collection (*n* = 11), in blue, individuals from the Piracicaba collection (*n* = 15), and in green, individuals from the Paraibuna collection (*n* = 50). **(B)** Discriminant analysis of principal components (DAPC) for 73 *E. brasiliensis* accessions collected in Natividade da Serra, Piracicaba and Paraibuna. The axis represents the first two main components of the Discriminant Analysis (DA). Each shape represents a site of collection and each color represents the different subpopulations identified by the DAPC analysis. **(C)** Graph representing the probability of membership of each individual from the three *E. brasiliensis* collections to specific genetic groups using the *K-means* method.

The analysis of molecular variance (AMOVA) disclosed that 29% of the total genetic variation is found between collections, while the remainder (71%) is found within collections (**Table 2**). The estimated *Phi_ST_* value for the *E. brasiliensis* collections was 0.29 (*p* < 0.001) (**Table 2**), indicating high genetic divergence between the three collections. Paired *Phi_ST_* estimates were high (**Table 3**), with values varying from 0.30 to 0.37, with the greatest divergence observed between the Piracicaba and Natividade da Serra collections (*Phi_ST_* = 0.37), and the smallest between Natividade and Paraibuna (*Phi_ST_* = 0.30). The high genetic divergence suggested by pairwise *Phi_ST_* estimates was also observed in the principal component analysis (PCA). The PCA analysis, based on 2299 SNPs, explained 34.8% of the total variation in the first two components and demonstrated that the *E. brasiliensis* collections are highly distinct from each other (**Figure 2A**).

**Table 2:** Analysis of molecular variance (AMOVA) considering the distribution of genomic diversity between and within the three collections of *Eugenia brasiliensis* (grumixama*)* accessions based on 2299 single nucleotide polymorphism (SNPs) markers; between and within the four collections of *E. pyriformis* (uvaia) accessions, based on 2872 SNPS; and between and within the seven collections of *E. involucrata* (Rio Grande cherry*)* accessions, based on 1471 single nucleotide polymorphism markers.

Source of variation	SS	MS	%	*Phi_ST_*
*Eugenia brasiliensis* (Grumixama)				
Between collections	10501.85	5250.93	29	0.29*
Within collections	45648.23	319.22	71	
*E. pyriformis* (Uvaia)				
Between collections	7800.86	2600.28	10	0.10*
Within collections	83628.96	459.50	90	
*E. involucrata* (Rio Grande cherry)				
Between collections	7738.25	1289.71	23	0.23*
Within collections	26009.91	222.31	77	

Sum of squares (SS); mean square (MS); percentage of variation (%); Wright fixation index (*Phi_ST_*); *significant at *p* < 0.001.

**Table 3:** Paired estimates of *Phi_ST_* (Weir and Cockerham, 1984) between collections of *Eugenia brasiliensis* (grumixama) accessions based on 2299 single nucleotide polymorphism markers.

Collections	Natividade da Serra	Piracicaba
Piracicaba	0.37*	
Paraibuna	0.30*	0.35*

*significant with *p* < 0.001.

The population structure and genetic relationships among the 73 *E. brasiliensis* accessions were also explored by discriminant analysis of principal components (DAPC), considering the optimal number of genetic groups (*K* = 6), determined by the *K-means* method (**Supplementary Figure S1**). The groups formed in the DAPC retained 81.3% of the total variation in the first two principal components (**Figure 2B**). The clusters formed showed great association with the collection of origin, and the grouping patterns were very similar to those observed by PCA (**Figure 2A**), reinforcing the high genetic structure found among the *E. brasiliensis* collections. The Paraibuna collection consisted of three genetic groups (2, 4, and 6), exhibiting substantial mixing. Conversely, the Piracicaba collection was composed of two distinct genetic groups (3 and 5). The Natividade da Serra collection contained only one genetic group (1) (**Figure 2C**).

#### 3.2.1 *Eugenia brasiliensis* core collections

To identify the smallest set of accessions that represent the available genetic diversity identified in this study for *E. brasiliensis*, three independent sample proportions were constructed (Collection 1, 2, and 3), with sizes varying from 10% to 30% of the entire dataset (**Table 4**). The largest sample, composed of 23 individuals (C3), managed to capture 99% of the 4598 alleles detected, while the smallest sample, C1, captured 4486 alleles, approximately 5% less than the total number of alleles detected. The C1 subcollection comprises one individual from Natividade da Serra, two from Piracicaba and five individuals from Paraibuna. C2 is made up of two individuals from Natividade da Serra, two from Piracicaba and 11 from Paraibuna. The C3 subcollection comprises two individuals from Natividade da Serra, two from Piracicaba, and 19 individuals from Paraibuna.

**Table 4:** Estimates of genetic diversity for the total number of individuals and proposed nuclear collections of *Eugenia brasiliensis* (grumixama), based on 2299 single nucleotide polymorphism markers.

Nuclear collection	*N*	*Na*	*Ho*	*He*	*f*	TDSC (%)
C1	8	4486	0.25	0.30	0.12	95
C2	15	4554	0.24	0.29	0.15	99
C3	23	4567	0.24	0.28	0.16	99
Total number of individuals	73	4598	0.24	0.30	0.19	100

Number of individuals (N); number of alleles (*Na*); observed (*Ho*) and expected (*He*) heterozygosities; fixation index (*f*); total SNP diversity captured (TDSC).

The genetic diversity indices obtained for the collections were similar or higher than those for the total number of individuals. *Ho* values ranged from 0.24 (C1 and C2) to 0.25 (C3), with the value for C3 being slightly above the value observed for all 73 accessions (0.24). *He* values varied from 0.28 in C3 to 0.30 in C1, which presented a *He* value equal to that of the total number of individuals (*He* = 0.30). The C1, C2, and C3 subcollections captured between 95% and 99% of the alleles, making them suitable for use in conservation and breeding.

**Figure 3:**
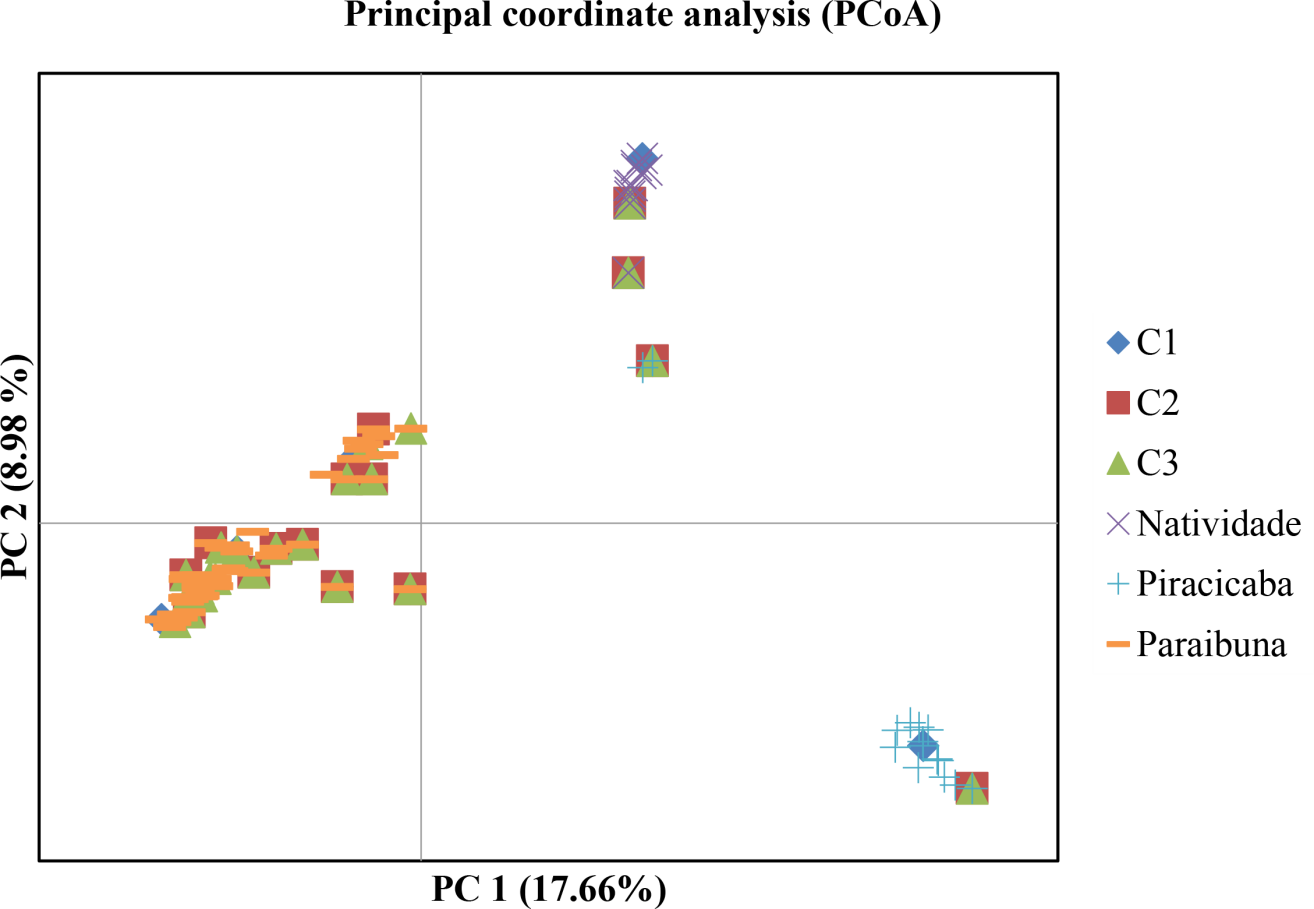
Principal coordinate analysis (PCoA), showing the dispersion of accessions from the three nuclear collections (C1, C2 and C3) and *Eugenia brasiliensis* (grumixama) accessions collected in Natividade da Serra, Piracicaba and Paraibuna.

The representativeness of the nuclear collections was also evaluated by principal coordinate analysis (PCoA), which showed the distribution of the 73 accessions collected in the three sampled locations (Natividade da Serra, Piracicaba and Paraibuna) and the core collection assembled from the 2299 SNP loci is along the first two coordinates (**Figure 3**). The variation explained by the first two coordinates was 26.6%, indicating a general overlap of diversity between the accessions selected for the core collections and those sampled in the three locations. This result suggests that any of the three core collections would adequately represent the total genetic diversity of *E. brasiliensis* identified in this study.

### 3.3 Population genomic analysis of *E. pyriformis*

We used the 2,872 identified SNPs to evaluate the genetic diversity and population structure of the 93 accessions from the four collections sampled in São Paulo and Minas Gerais. The genetic diversity (*He* and *Ho*) was similar between the *E. pyriformis* collections (**Table 1**). *He* ranged from 0.18 (Inconfidentes and Esalq) to 0.22 (Cabo Verde), with an average of 0.19. The observed heterozygosity (*Ho*) average was 0.18, and ranged from 0.15 (Esalq) to 0.22 (Cabo Verde). The highest number of private alleles (*Pa*) was observed for the Cabo Verde collection (193), and the lowest for the Rio Claro collection, which did not contain any private alleles. The Cabo Verde collection also exhibited the highest percent of polymorphic loci (%*P*), number of alleles per locus (*A*), and number of alleles (*Na*). In the other collections, genetic diversity estimates were similar, with %*P* varying from 67.5% (Inconfidentes) to 72.8% (Rio Claro), *Na* ranging from 1.67 (Inconfidentes) to 1.72 (Rio Claro), and *A* being the same for all three locations. Regarding the fixation index (*f*), the Cabo Verde and Esalq collections presented positive values and significantly different from zero, indicating the presence of inbreeding (*f* = 0.09 and *f* = 0.14, respectively). The Rio Claro collection (*f* = 0.06) also showed a positive fixation index; but it was not significant. The Inconfidentes collection presented *f* = -0.08 (with a 95% confidence interval not including zero), indicating a partiality for heterozygotes in this collection.

The analysis of molecular variance revealed that the largest proportion of genetic variation was found within the *E. pyriformis* collections (**Table 2**), with moderate divergence between the collections, with *Phi_ST_* = 0.10 (*p* < 0.001). Paired *Phi_ST_* estimates between *E. pyriformis* collections indicate a moderate to high level of genetic divergence (**Table 5**). The largest divergence was observed between the Esalq and Inconfidentes collections (*Phi_ST_* = 0.26), and the smallest, between the Cabo Verde and Rio Claro collections (*Phi_ST_* = 0.06).

**Table 5:** Paired *Phi_ST_* estimates (Weir and Cockerham, 1984) between collections of *Eugenia pyriformis* (uvaia) accessions based on 2872 single nucleotide polymorphism markers.

Collections	Rio Claro	Cabo Verde	Inconfidentes
Cabo Verde	0.06		
Inconfidentes	0.16	0.15	
Esalq	0.15	0.17	0.26

**Figure 4:**
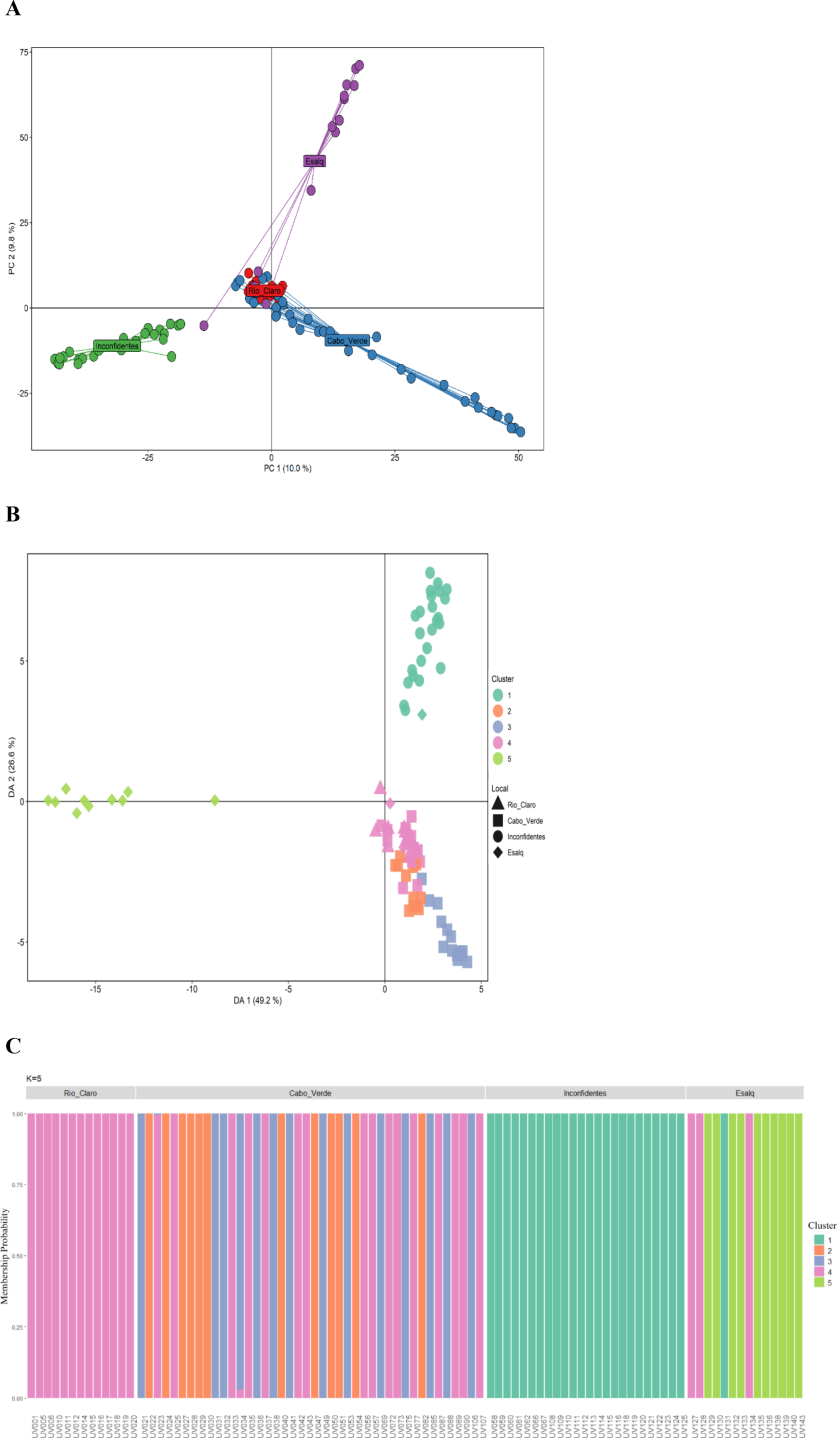
**(A)** Principal component analysis (PCA) representing the genetic structure of four *Eugenia pyriformis* (uvaia) collections, based on 2872 single nucleotide polymorphism markers. In red individuals from the Rio Claro collection (*n* = 16), in purple the Esalq collection (*n* = 20), in blue the Cabo Verde collection (n = 53) and in green individuals from the Inconfidentes collection (*n* = 22). **(B)** Discriminant analysis of principal components (DAPC) for 93 *E. pyriformis* accessions collected in Rio Claro, Cabo Verde, Inconfidentes and Esalq. The axis represent the first two main components of the Discriminant Analysis (DA). Each shape represents a source collection and each color represents the different groups identified by the DAPC. **(C)** Graph representing the probability of membership of each individual from the four *E. pyriformis* collections to the genetic groups determined by the *K-means* method.

The genetic structure analysis, performed using PCA based on the 2872 SNPs, explained 19.8% of the total variation in the first two principal components (**Figure 4A**). This analysis agreed with the paired *Phi_ST_* estimates, as it is possible to observe greater genetic divergence between the Esalq and Inconfidentes collections compared to the other collections, and that individuals from Cabo Verde and Rio Claro overlapped, justifying the smaller divergence between these collections. Although individuals from the Esalq collection showed some overlap with the Rio Claro collection, they are still very distinct.

The optimal number of genetic groups estimated by the *K-means* method within the set of 93 *E. pyriformis* accessions was *K* = 5 (**Supplementary Figure S2**). In this way, five groups were plotted using DAPC, accounting for 75.8% of the total variation in the first two principal components (**Figure 4B**). Scatter plot analysis indicated the formation of three arbitrary clusters, in which groups 1 and 5 are more easily distinguishable than groups 2, 3, and 4, which overlap. This analysis, as well as the PCA, also showed greater genetic divergence between the Inconfidentes and Esalq collections, since the Inconfidentes´ accessions were all assigned to group 1, while those from Esalq were mostly assigned to group 5 by DAPC. Likewise, the Cabo Verde and Rio Claro accessions were assigned to groups 2, 3 and 4 (**Figure 4B**). The probability of adherence for each individual ranged from 95 to 100%, indicating that the identified genetic groups showed little mixing (**Figure 4C**).

### 3.4 Population genomic analysis of *E. involucrata*

We utilized the 1,471 SNP markers identified by GBS to assess genetic diversity and population structure in the 62 *E. involucrata* accessions collected across seven collections. The average value of *He* and *Ho* for the seven collections was 0.21 and 0.22, respectively. Jundiaí 1 showed the highest genetic diversity (*He* = 0.26 and *Ho* = 0.31), while Piracicaba 1 showed the lowest (*He* = 0.11 and *Ho* = 0.14) (**Table 1**). The average number of alleles per locus (*A*) was 1.35. The lowest *A* was found in Piracicaba 1 (1.19) and the highest in Jundiaí 1 (1.44). The number of alleles (*Na*) ranged from 1.21 (Piracicaba 1) to 1.53 (Jundiaí 1), with an average of 1.66. The Jundiaí 1 and 2 and Piracicaba 2 collections did not present any private allele. The highest *Pa* was observed in Piracicaba 1 (*Pa* = 5). The average fixation index (*f*) for all seven collections was -0.04, indicating a relatively low overall level of inbreeding. The fixation indices were negative for the Piracicaba 1, Jundiaí 1, and Jundiaí 2 collections (*f* = -0.22, -0.16, and -0.34, respectively), suggesting an excess of heterozygotes in these collections. The collections from Rio Claro, Piracicaba 2, Inconfidentes and Paraibuna presented non-significant f values.

**Figure 5:**
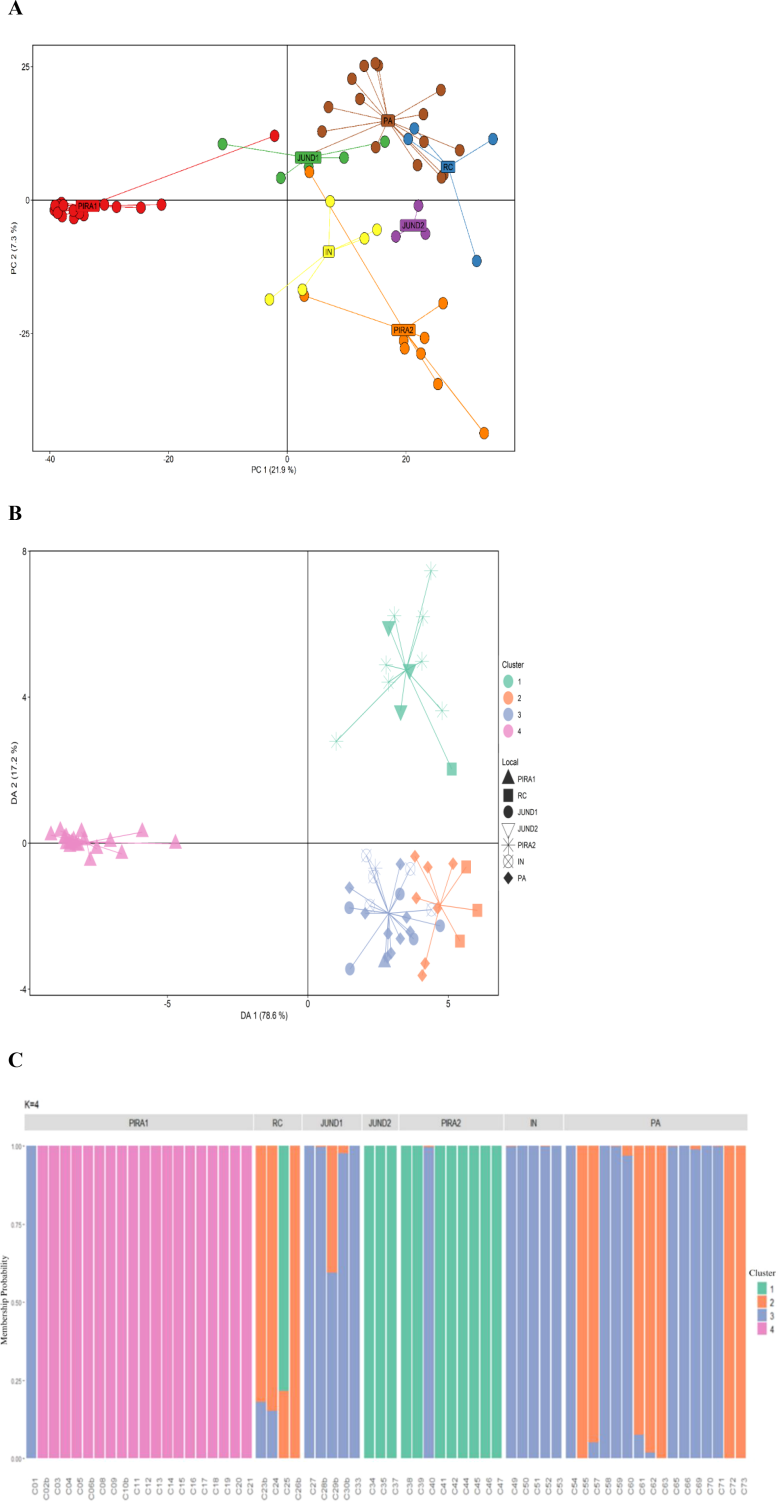
**(A)** Principal component analysis (PCA) representing the genetic structure of seven *Eugenia involucrata* (Rio Grande cherry) collections based on 1471 single nucleotide polymorphism markers. In red individuals from the collection of Piracicaba 1 (PIRA1, n = 21) and in orange from Piracicaba 2 (PIRA2, *n* = 10), in green Jundiaí 1 (JUND1, *n* = 5), in purple Jundiaí 2 (JUND2, *n* = 4), in blue Rio Claro (RC, *n* = 4), in yellow Inconfidentes (IN, *n* = 5) and in brown individuals from the Paraíbuna collection (PA, *n* = 20). **(B)** Discriminant analysis of principal components (DAPC) for 62 *E. involucrata* accessions collected from seven different locations. The axis represent the first two main components of the Discriminant Analysis (DA). Each shape represents a source collection and each color represents the different subpopulations identified by the DAPC analysis. **(C)** Graph representing the probability of membership of each individual from the seven *E. involucrata* collections to the genetic groups determined by the *K-means* method.

The AMOVA results revealed genetic differentiation among *E. involucrata* collections, with *Phi_ST_* = 0.23, representing 23% of the total variation, with most variation occurring within collections (**Table 2**). *Phi_ST_* values between collections ranged from 0.07 to 0.57, indicating a moderate to high level of genetic divergence (**Table 6**). All comparisons between the Piracicaba 1 collection and the other collections showed the highest *Phi_ST_* values, indicating a higher level of genetic divergence in this collection in relation to the others. This divergence is best visualized by PCA (**Figure 5A**), where a clear separation of the Piracicaba 1 collection from the other collections is noticeable.

**Table 6:** Paired *Phi_ST_* estimates (Weir and Cockerham, 1984) among 62 *Eugenia involucrata* (Rio Grande cherry) accessions based on 1471 single nucleotide polymorphism markers.

Collections	Piracicaba 1	Rio Claro	Jundiaí 1	Jundiaí 2	Piracicaba 2	Inconfidentes
Rio Claro	0.57					
Jundiaí 1	0.36	0.09				
Jundiaí 2	0.57	0.13	0.14			
Piracicaba 2	0.52	0.12	0.15	0.14		
Inconfidentes	0.45	0.15	0.09	0.16	0.14	
Paraibuna	0.47	0.07	0.09	0.13	0.17	0.15

The clustering based on the K-means method showed *K* = 4 (**Supplementary Figure S3**). The four groups formed in DAPC based on the K-means retained 95.8% of the total variation in the first two principal components for the 62 *E. involucrata* accessions (**Figure 5B**). In the scatterplot of the DAPC analysis, it is possible to observe that some groups, such as groups 1 and 4, are more easily distinguishable than others. Group 4 was formed exclusively by individuals from the Piracicaba 1 collection, reinforcing the greater genetic divergence suggested by the *Phi_ST_* and PCA values. Group 1 was formed by individuals from the Jundiaí 2 and Piracicaba 2 collections, that also showed greater genetic divergence in relation to the others. There is also a small overlap of individuals between groups 2 and 3, showing less genetic divergence between these groups, which were formed by individuals from the Rio Claro and Paraibuna collections (group 2), and by individuals from Jundiaí 1, Inconfidentes and Paraibuna (group 3). The association between the origin of the access (collection) and the four genetic groups formed can be better visualized by the probability graph of individuals joining each group (**Figure 5C**).

## 4 Discussion

This is a pioneering study on the genetic diversity of three Eugenia species, *E. brasiliensis* (grumixama), *E. pyriformis* (uvaia), and *E. involucrata* (Rio Grande cherry), which are promising new fruit crops, based on SNP markers. GBS has been successfully used to discover SNPs for studies of genomic diversity and population structure in a wide range of species, such as *Camelina sativa* (Luo et al., 2019), *Capsicum* spp (Pereira-Dias et al., 2019), *Phaseolus vulgaris* (Delfini et al., 2021), *Acrocomia* spp (Díaz et al., 2021), *Psidium guajava* (Diaz-Garcia and Padilla-Ramírez, 2023) and *Elaeis oleifera* (Kunth) Cortés (Leão et al., 2022) to name a few. Here, GBS was used to identify SNP markers and evaluate the patterns of genomic diversity and population structure of 73 *E. brasiliensis* accessions, 93 *E. pyriformis* accessions, and 62 *E. involucrata* accessions, maintained by research institutions and small growers, used for urban afforestation and production in São Paulo and Minas Gerais. The number of SNPs revealed ranged from 1,471 for *E. pyriformis* to 2872 SNPS for *E. pyriformis*, a large number of markers to use in genetic diversity studies.

The analyses revealed that the average level of genetic diversity (*He*) was similar among the three species (**Table 1**). The *He* values obtained in this study are lower than those reported by Ferreira-Ramos et al. (2014), who genotyped 26 *E. pyriformis* accessions and 16 *E. brasiliensis* accessions using microsatellite markers, and with average values of 0.75 and 0.54 for *He*, respectively. Stefanel et al. (2021) also found higher *He* values (0.62) for *E. involucrate*, using microsatellite markers. When the *He* values observed in this study are compared with those reported for other Myrtaceae species genotyped using SNP markers, the discrepancies between the estimates are reduced. For example, *He* = 0.22 for *Eucalyptus camaldulensis* (Dillon et al., 2014) and *He* = 0.30 for *E. urophylla* (Yang et al., 2020). Similar, *He* was also reported by Izuno et al. (2017) for *Metrosideros polymorpha*, which observed *He* varying from 0.19 to 0.25 using SNPs. Such discrepancies are expected and can be explained by considering the nature of the markers: SSRs are multiallelic and more polymorphic than biallelic SNP markers (Singh et al., 2013). On the other hand, SNPs occur at a much higher density throughout the genome and have lower genotyping error rates (Hamblin et al., 2007). Furthermore, SNPs more accurately reflect actual patterns of genetic diversity and genome structure than microsatellite markers (Telfer et al., 2015; Silva et al., 2020).

The observed mean heterozygosity (*Ho*) for the *E. brasiliensis* (*Ho* = 0.25) and *E. involucrata* (*Ho* = 0.22) collections were slightly higher than the *He* found for these species. For the *E. pyriformis* collections, the value (*Ho* = 0.18) was slightly lower (**Table 1**). Excess heterozygotes in *E. brasiliensis* and *E. involucrata* were also reported in previous studies using SSRs (Ferreira-Ramos et al., 2014; Stefanel et al., 2021). In the case of *E. pyriformis*, a low level of homozygotes was also reported by Ferreira-Ramos et al. (2014). These results are consistent with the *f* values observed in this study. The fixation index (*f*) is one of the most important parameters in population genetics, as it measures the balance between homozygotes and heterozygotes in populations. The average *f* found for each of the species evaluated was -0.06 for the three *E. brasiliensis* collections, 0.05 for the four *E. pyriformis* collections, and -0.04 for the seven *E. involucrata* collections (**Table 1**). The relatively low values of inbreeding found may be attributed to the higher rate of cross-fertilization in these species. Although there are no detailed studies on the mating system of *E. brasiliensis*, *E. pyriformis*, or *E. involucrata*, the occurrence of higher rates of cross-fertilization has been reported for other species of the genus *Eugenia*, such as *E. uniflora, E. punicifolia, E. neonitida*, and *E. rotundifolia* (Silva and Pinheiro, 2009; Diniz and Buschini, 2016). Additionally, the fact that bees are the most important pollinators of these species (Gressler et al., 2006) may, in part, explain the relatively low level of inbreeding found.

Furthermore, the population differentiation found by AMOVA indicated greater variation within collections (**Table 2**), a common characteristic in cross-pollinated plants, which maintains their genetic variability distributed within populations, suggesting the predominance of cross-hybridization in these species. Sample size and/or genetic drift could also explain the negative fixation index found in some collections. However, other hypotheses for negative values of *f* can be highlighted, including the mixing of previously isolated populations and/or the presence of hybrids (Zalapa et al., 2010), negative selective mating, when reproduction occurs between individuals with more different phenotypes than by chance (Stoeckel et al., 2006), and unconscious selection of more heterozygous individuals to form collections.

A high number of private alleles (*Pa*) was observed in the *E. brasiliensis* and *E. pyriformis* collections, with emphasis on the Paraibuna (*E. brasiliensis*) and Cabo Verde (*E. pyriformis*) collections, which presented the highest number of private alleles (**Table 1**). The presence of many private alleles in these collections may be attributed to genetic drift and the absence of gene flow resulting from isolation. Another hypothesis to be considered in relation to the *Pa* rate is that, as these are managed collections, their genetic constitution may be altered by human-made introductions. The *E. involucrata* collections showed few and/or no private alleles (**Table 1**). The low number of private alleles in *E. involucrata* collections must be related to the restricted number of individuals that make up such collections.

AMOVA revealed that most of the genetic variation was found within the collections for all three species: 71% for *E. brasiliensis*, 77% for *E. involucrata*, and 90% for *E. pyriformis*. The remaining variation (29%, 23%, and 10%, respectively) was observed among collections within each species (**Table 2**). These results are consistent with the *Phi_ST_* values obtained (*Phi_ST_* = 0.29, 0.23, and 0.10, respectively; *P* > 0.01), which indicate moderate to very high genetic differentiation between collections. According to Hartl and Clark (2010), *Phi_ST_* values can be classified into four categories: low differentiation (0–0.05), moderate (0.05–0.15), high (0.15–0.25), and very high (>0.25). The observed patterns are in line with previous findings for Myrtaceae species, including high within-population genetic variation in *E. involucrata* based on RAPD markers (Rejane et al., 2022), and similar results for *Campomanesia phaea* (Moreira et al., 2022), *Myrciaria floribunda* (Franceschinelli et al., 2007), and *Eugenia dysenterica* (Zucchi et al., 2003).

The moderate to high *Phi_ST_* values suggest limited historical gene flow between the collections, likely due to geographic isolation and restricted genetic exchange. It is important to note that *Phi_ST_* reflects the cumulative effects of past gene flow and does not capture contemporary reproductive events (Smouse and Sork, 2004). Additionally, since these are human-managed collections, the observed genetic structure may also reflect selective sampling, particularly from a small number of mother trees with desirable phenotypic traits. This may explain the modest admixture observed in some collections, such as Paraibuna (*E. brasiliensis*), Cabo Verde (*E. pyriformis*), Rio Claro, Jundiaí 1, and Paraibuna (*E. involucrata*).

Overall, the results indicate strong genetic structuring among the *E. brasiliensis*, *E. pyriformis*, and *E. involucrata* collections, despite moderate levels of genetic diversity within them. These patterns, confirmed by Wright’s F statistics as well as PCA and DAPC analyses, highlight the potential of these genetic resources for both conservation programs and future breeding initiatives targeting traits of agronomic interest.

The main objective of developing a core collection is to capture the maximum genetic diversity present in the germplasm in a reduced set of accessions (Egan et al., 2022). In this study, the first effort was made to create a core collection of *E. brasiliensis* accessions. To achieve the goal of representing at least 70% of the diversity present in the total sample, three possible basic *E. brasiliensis* collections were proposed (Odong et al., 2013). Nuclear collections account for more than 95% of the total allelic variability in sampled individuals. Furthermore, the proposed nuclear collections yielded genetic diversity estimates similar to those of the total individuals, indicating a good representation of these collections. These core collections may serve as a foundation for future studies focused on fruit quality traits and could be strategically used to initiate genetic breeding programs for the species at a lower maintenance cost.

In summary, our study is the first to evaluate the genetic diversity of *E. brasiliensis* (grumixama), *E. pyriformis* (uvaia), and *E. involucrata* (Rio Grande cherry) using SNP markers obtained by NGS technologies. As highlighted by Willing et al. (2012), the use of an extensive set of biallelic genetic markers is suitable for investigating genetic diversity, even when population samples are limited, as is the case in this study. The identified SNPs proved to be valuable even for *E. involucrata*, where sample collection was restricted in some locations and in a small geographic area. In general, the genetic diversity observed in this study tended to be lower than that reported in previous studies using second-generation markers, such as SSR, in *Eugenia* species. However, this discrepancy was expected, given that SNP markers, predominantly biallelic, have lower mutation rates than SSRs. On the other hand, SNPs are distributed at a higher density throughout the genome and are less prone to genotyping error, thus enabling more robust estimates of genetic diversity and genetic structuring.

The application of genetic evaluation based on NGS has expanded our understanding of the genetic diversity and population structure of these species, providing the basis for building a core collection for *E. brasiliensis* and opening new perspectives to assist future phenotyping studies in this species. *Eugenia brasiliensis* (grumixama), *E. pyriformis* (uvaia) and *E. involucrata* (Rio Grande cherry) stand out for their considerable economic potential. Therefore, the use of these population genomics data can be fundamental in defining best management practices for these species, to preserve and value their genetic resources.

## Data Availability

The datasets presented in this study can be found in online repositories. The names of the repository/repositories and accession number(s) can be found below: https://www.ebi.ac.uk/biostudies/studies/S-BSST2086?key=58c4f841-6648-4740-b7b1-1f28747ac341, S-BSST2086 https://www.ebi.ac.uk/biostudies/studies/S-BSST2087?key=22c49bcb-5085-4bfd-9dc4-866d4927bf7b, S-BSST2087 https://www.ebi.ac.uk/biostudies/studies/S-BSST2088?key=b5279de8-b15f-4ca7-8bd1-e45dd2f3526e, S-BSST2088.

## Supplementary material

Supplementary Table 1 Location and number of accessions collected (*n*) of *Eugenia brasiliensis* (grumixama), *E. pyriformis* (uvaia) and *E. involucrata* (Rio Grande cherry). States: SP: São Paulo; MG: Minas Gerais.

Supplementary Figure 1 Determination of the optimal number of genetic clusters (K) for *Eugenia brasiliensis* (grumixama). The Bayesian Information Criterion (BIC) is plotted against the number of potential clusters (K). The analysis was based on 73 accessions and 2,299 SNPs. The lowest BIC value (404.0) indicates the optimal number of clusters, determined as K = 6.

Supplementary Figure 2 Determination of the optimal number of genetic clusters (K) for *Eugenia pyriformis* (uvaia). The Bayesian Information Criterion (BIC) is plotted against the number of potential clusters (K). The analysis was based on 93 accessions and 2,872 SNPs. The lowest BIC value indicates (533.2) the optimal number of clusters, determined as K = 5.

Supplementary Figure 3 Determination of the optimal number of genetic clusters (K) for *Eugenia involucrata* (Rio Grande cherry). The Bayesian Information Criterion (BIC) is plotted against the number of potential clusters (K). The analysis was based on 62 accessions and 1,471 SNPs. The lowest BIC value (326.1) indicates the optimal number of clusters, determined as K = 4.
