# Supplementary material for: Population genomics of Brazilian native fruit species of Eugenia spp. (Myrtaceae) for conservation and improvement
Source: Front Plant Sci. 2026 Jan 12;16:1670349. doi: 10.3389/fpls.2025.1670349 (PMC12836885; doi:10.3389/fpls.2025.1670349)
Supplement: Supplementary Table 1 — Location and number of accessions collected (n) of Eugenia brasiliensis (grumixama), E. pyriformis (uvaia) and E. involucrata (Rio Grande cherry). States: SP: São Paulo; MG: Minas Gerais. [file DataSheet1.docx]

Population genomics of Brazilian native fruit species of *Eugenia* spp. (Myrtaceae) for conservation and improvement

Laecio Fernandes Souza Sampaio^1^, Maria Imaculada Zucchi^2^, Carlos Augusto Colombo^3^, Angelo Pedro Jacomino^1^, Antonio Figueira^4^, Francisco de Assis Alves Mourão Filho^1*^

^1^Escola Superior de Agricultura Luiz de Queiroz, Universidade de São Paulo, Piracicaba, SP, Brazil

^2^Polo Regional de Desenvolvimento Tecnológico do Centro Sul, Agência Paulista de Tecnologia dos Agronegócios, Piracicaba, SP, Brazil

^3^Instituto Agronômico de Campinas, Centro de Recursos Genéticos Vegetais, Campinas, SP, Brazil.

^4^Centro de Energia Nuclear na Agricultura, Universidade de São Paulo, Piracicaba, SP, Brazil

*** Correspondence:** francisco.mourao@usp.br

Supplementary Table S1. Location and number of accessions collected (*n*) of grumixama (*Eugenia brasiliensis*), uvaia (*E. pyriformis*) and Rio Grande cherry (*E. involucrata*).

| Species | Municipality, State | Site | Latitude | Longitude | *n* |
| --- | --- | --- | --- | --- | --- |
| *Eugenia brasiliensis* | Natividade da Serra - SP | Sítio Paulo Nakanishi | 23° 31.519'S | 45° 29.750'W | 11 |
|  | Piracicaba - SP | Faz. Areão (Esalq) | 22° 41.529'S | 47° 38.693'W | 15 |
|  | Paraibuna -SP | Sítio do Belo | 23° 27.773'S | 45° 42.562'W | 50 |
| **Total** |  |  |  |  | **76** |
| *Eugenia pyriformis* | Rio Claro – SP | Sítio Sartori | 22° 15.912'S | 47° 32.675'W | 16 |
|  | Piracicaba – SP | Faz. Areão (Esalq) | 22° 41.529'S | 47° 38.693'W | 20 |
|  | Cabo Verde – MG | Faz. Renato | 21° 30.626'S | 46° 20.230'W | 53 |
|  | Inconfidentes - MG | IFSULMINAS | 22° 18.612'S | 46° 19.761'W | 22 |
| **Total** |  |  |  |  | **111** |
| *Eugenia involucrata* | Piracicaba 1 - SP | Faz. Areão (Esalq) | 22° 41.529'S | 47° 38.693'W | 21 |
|  | Piracicaba 2 - SP | R. Prudente de Morais | 22° 43.567'S | 47° 38.562'W | 10 |
|  | Inconfidentes - MG | IFSULMINAS | 22° 18.612'S | 46° 19.761'W | 5 |
|  | Paraibuna - SP | Sítio do Belo | 23° 27.773'S | 45° 42.562'W | 20 |
|  | Rio Claro - SP | Sítio Sartori | 22° 15.912'S | 47° 32.675'W | 4 |
|  | Jundiaí 1 – SP | Beira de Estrada | 23° 07.363'S | 46° 57.579'W | 5 |
|  | Jundiaí 2 - SP | Beira de Estrada | 23° 15.470'S | 46° 52.808'W | 4 |
| **Total** |  |  |  |  | **69** |

States: SP: São Paulo; MG: Minas Gerais.


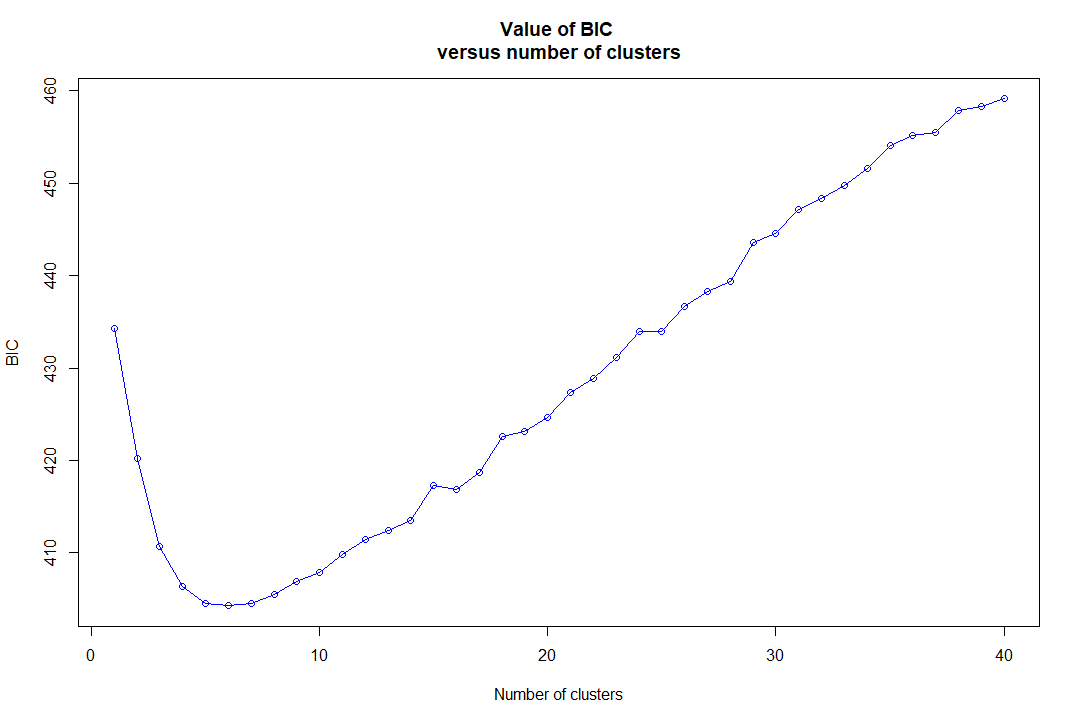
Supplementary Figure S1. Determination of the optimal number of genetic clusters (K) for *Eugenia brasiliensis* (grumixama). The Bayesian Information Criterion (BIC) is plotted against the number of potential clusters (K). The analysis was based on 73 accessions and 2,299 SNPs. The lowest BIC value (404.0) indicates the optimal number of clusters, determined as K = 6.


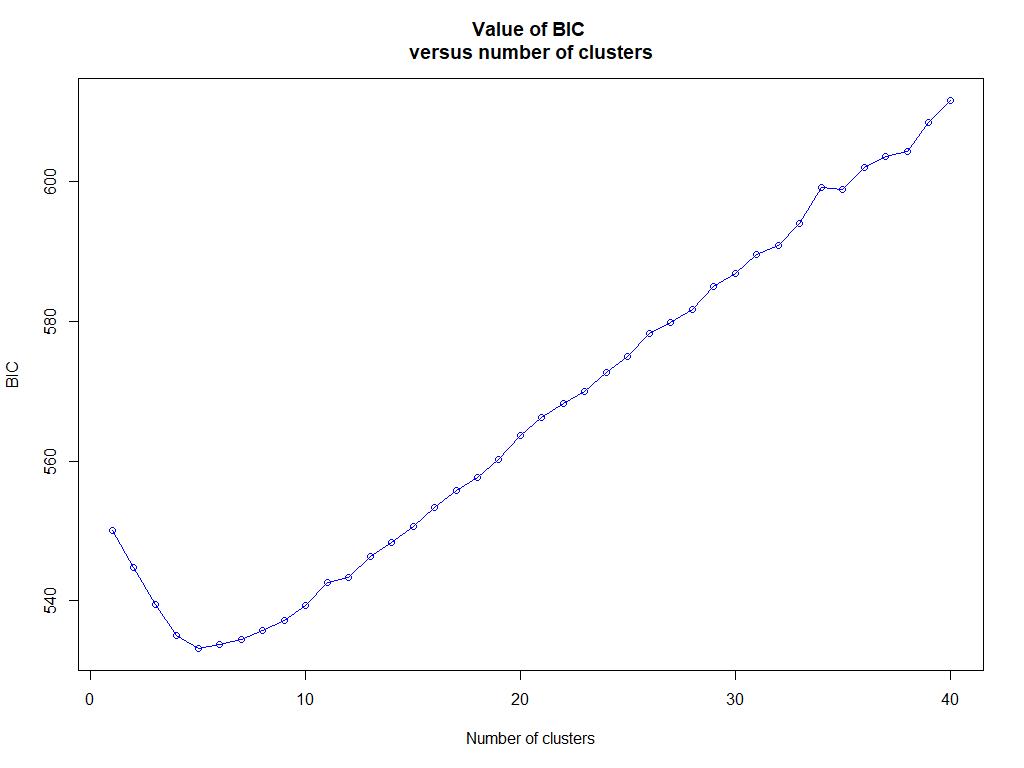
Supplementary Figure S2. Determination of the optimal number of genetic clusters (K) for *Eugenia pyriformis* (uvaia). The Bayesian Information Criterion (BIC) is plotted against the number of potential clusters (K). The analysis was based on 93 accessions and 2,872 SNPs. The lowest BIC value indicates (533.2) the optimal number of clusters, determined as K = 5.


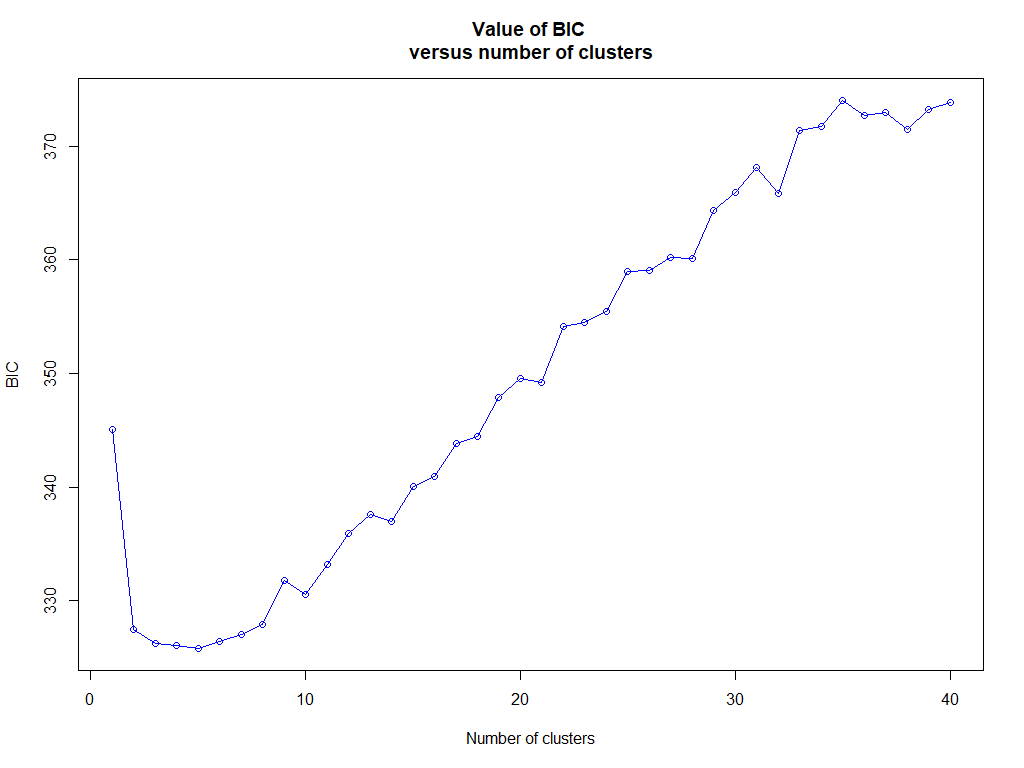
Supplementary Figure S3. Determination of the optimal number of genetic clusters (K) for *Eugenia involucrata* (Rio Grande cherry). The Bayesian Information Criterion (BIC) is plotted against the number of potential clusters (K). The analysis was based on 62 accessions and 1,471 SNPs. The lowest BIC value (326.1) indicates the optimal number of clusters, determined as K = 4.
